# Application of the Team Emergency Assessment Measure Scale in undergraduate medical students and interprofessional clinical teams: validity evidence of a Spanish version applied in Chile

**DOI:** 10.3389/fmed.2023.1256982

**Published:** 2023-09-13

**Authors:** Soledad Armijo-Rivera, Sandra Ferrada-Rivera, Marcela Aliaga-Toledo, Leonardo A. Pérez

**Affiliations:** ^1^Escuela de Medicina, Facultad de Medicina Clínica Alemana Universidad del Desarrollo, Santiago, Chile; ^2^Unidad de Calidad y Seguridad del Paciente, Hospital Padre Hurtado, Santiago, Chile; ^3^Departamento de Desarrollo de las Personas, Hospital Padre Hurtado, Santiago, Chile; ^4^Centro de Habilidades Clínicas, Facultad de Medicina, Universidad de Chile, Santiago, Chile; ^5^Centro de Habilidades Clínicas y Disciplinares, Universidad de O'Higgins, Rancagua, Chile

**Keywords:** teamwork, leadership, interprofessional simulation, emergency, medical education

## Abstract

**Background:**

Teamwork is one of the competencies necessary for physicians to work effectively in health systems and is a competency that can be developed with simulation in professionals and medicine students. The Team Emergency Assessment Measurement (TEAM) was created to evaluate the non-technical performance of team members during resuscitation events in real teams. The TEAM scale includes items to assess leadership, teamwork, situational awareness, and task management. An objective evaluation tool in Spanish is valuable for training health professionals at all undergraduate and continuing education levels. This study aimed to generate evidence of the validity of the Team Emergency Assessment Measure (TEAM) in Spanish to measure the performance of medical students and adult, pediatric, and obstetric emergency clinical teams in simulated emergencies as a self-assessment tool.

**Methods:**

To develop the Spanish version of the instrument, a forward and backward translation process was followed by independent translators, native and fluent in English and Spanish, and a review by a panel of Chilean experts comprising three trained simulation instructors to verify semantics and cultural equivalence. High-fidelity simulations with debriefing were conducted with 5th-year medical students, in which students and instructors applied the Spanish version of the TEAM scale. In the second stage, adult, pediatric, and obstetric emergency management simulations were conducted using the TEAM scale for real clinical teams as a self-assessment tool.

**Findings:**

By applying the overall TEAM scale to medicine students and clinical teams, Cronbach's alpha was 0.921. For medical students' self-assessment, we obtained Cronbach's alpha of 0.869. No significant differences were found between the overall scores and the scores by dimensions evaluated by instructors and students (*p* > 0.05). In the case of clinical team training, Cronbach's alpha was 0.755 for adult emergency teams, 0.797 for pediatric emergency teams, and 0.853 for obstetric emergency teams.

**Conclusion:**

The validated instrument is adequate for evaluating teamwork in medical student simulations by instructors and peers and for self-assessment in adult, pediatric, and obstetric emergency clinical teams.

## Introduction

At the beginning of the 21^st^ century, medical training required new means of assessing clinical reasoning, professionalism, and teamwork, among other competencies beyond the cognitive domain (1). In the Chilean context, the Association of Medical Schools (ASOFAMECH) recently recognized that “working efficiently in an interdisciplinary team, assuming leadership when required” is one of the competencies necessary for physicians to work effectively in health systems (2).

An analysis of the efficiency of curricula in teaching and evaluating teamwork found few reports on curricular insertions of teamwork competency. It showed that the effects of the interventions analyzed were low in the short term, recommending improvements in educational and evaluation practices for this competency (3).

On the other hand, a systematic review of the effects of advanced life support courses showed that the impact on patients in the short term is modest and recommends strengthening the development of teamwork skills (4).

One of the instruments described in the literature to assess teamwork is the Team Emergency Assessment Measure (TEAM), created for clinical experts to perform an accurate assessment and feedback on leadership skills, teamwork, situational awareness, and task management in real teams, and which has high levels of reliability in its original English version (5). Along with this, the TEAM scale in its English version has evidence of content, construct, and concurrent validity, as well as various reliability shreds of evidence derived from measurements of its internal consistency, inter-observer agreement, and test-retest agreement, derived from five studies developed in high-income Western countries and real teams (6).

In its English version, the TEAM scale has been used to evaluate the teamwork of medical and nursing students and registered nurses in the context of simulation training. It has also been used to evaluate video recordings of emergency teams of adult patients (7). In these cases, the instrument has been applied by expert clinicians rather than by the team members themselves.

This scale has also been used to assess team dynamics in adult general emergency (8), surgery (9), anesthesia (10), obstetric emergency (11), and pediatric emergency teams (12).

The original TEAM scale has reported versions in French (13), Finnish (14), German (15), Swedish (16), and Brazilian Portuguese (17). The author's website (http://medicalemergencyteam.com/) offers other versions in Hebrew, traditional and simplified Chinese, Spanish, Italian, Portuguese (from Portugal), Persian, and Korean. To date, there are neither reports of validated versions in Spanish, nor are there any reports comparing the results of the application of instructors and students in undergraduate medical contexts or the self-assessment in real teams.

A leadership and teamwork assessment instrument widely used globally and validated in Spanish is essential to guide the development of this professional competency throughout the training cycle of health professionals.

Generating evidence of validity for an instrument that allows the unified measurement of teamwork in the emergency department, from undergraduate to the labor field, can offer an opportunity to measure the transfer of competencies developed at the initial formative levels of health careers. Using it as a self-assessment tool in the undergraduate curriculum and real teams adds utility to the instrument and can help strengthen the culture of teamwork in healthcare.

This research aimed to generate evidence of the validity of the TEAM in Spanish to measure the performance of medical students and adult, pediatric, and obstetric emergency clinical teams in simulated emergencies as a self-assessment tool.

## Methods

We designed a quantitative assessment instrument validation study. Authorization was requested from the author of the TEAM scale to use the original instrument in English in Chile.

To develop this Spanish version of the scale, a forward and backward translation process was followed by independent translators, native and fluent in English and Spanish, and a review by a panel of Chilean experts constituted by three trained simulation instructors to verify semantics and cultural equivalence, following the Consensus-based Standards for the selection of health Measurement INstruments (COSMIN) (18).

The scale was applied in undergraduate education settings to medical students during the first semester of 2021, continuing interprofessional education to real clinical teams during the second semester of 2021.

The “Integrated Medical-Surgical III” is a fifth-year medical course at the Universidad del Desarrollo. The medical curriculum at Universidad del Desarrollo begins using standardized patient simulations in the third year to develop history-taking and physical examination abilities. During the fourth year, the students participate in high-fidelity simulations on managing prevalent pathologies in emergency settings (myocardial infarction, respiratory failure, etc.). At the end of both courses, a summative OSCE is performed. Students are familiarized with high-fidelity simulation and debriefing before reaching the 5^th^ year of their careers. Within the 5th-year course in which the intervention was carried out, the simulations analyzed correspond to those of the 3rd week of a 5-week course. This week was chosen so that the students could integrate well into the dynamics of the social group with which the simulation intervention and the study would take place.

We run six face-to-face simulation sessions for clinical reasoning, teamwork, and communication concerning patient handover. The students received study material for these general competency domains before the simulations. They were distributed in 18 groups of 4–5 students (82 students). On the second day of activity, two high-fidelity simulation scenarios of adult infectious pathologies evolving with a medical crisis were performed (Table 1). Two students acted in the roles of physicians who worked in a sequential leadership role in the scenario, while the other students acted in the roles of healthcare team members (nurses and paramedical technicians). Two instructors guided the scenarios face-to-face, one of them with CHSE-A (Certified Healthcare Simulation Educator Advanced) recognition. We used a SimMom™ simulator with a vital signs monitor, both controlled with the LLEAP™ software. After each scenario, the instructors performed co-debriefing in “follow the leader” (19). At the end of the session, the instructors and students who voluntarily agreed to participate in the study independently evaluated the teamwork in the two scenarios, applying the TEAM scale supported by the SurveyMonkey™ platform. The instrument's reliability was analyzed using Cronbach's alpha and the inter-observer agreement with a two-tailed Mann–Whitney non-parametric test. Ethics Committee approval was obtained (CEII 46/2018).

**Table 1:** Summary of characteristics of the programs evaluated.

**Program participants (population)**	**Size of participating teams**	**Scenarios**	**Debriefing**
18 Teams of fifth-year medical students.	4–5	- Hip arthritis presenting anaphylaxis to antibiotic. - Meningitis progressing to convulsive status.	Post-scenario “follow-the-leader” co-debriefing.
8 Pediatric emergency clinical teams 12 Pediatricians, 16 Nurses, 4 Kinesiologists, 16 Paramedical technicians, 8 Support assistants.	7	- Thoracic trauma, penetrating injury. - ECT and hypovolemic shock, automobile accident. - Abdominal trauma, gunshot wound. - Pelvis trauma and spinal shock, fall from height.	Microdebriefing during scenarios and post-simulation GAS debriefing.
5 Obstetric emergency clinical teams 10 Gynecologists, 6 Anesthesiologists, 13 Midwives, 8 Paramedical technicians, 2 Support assistants.	8	- Ovular remnants - Uterine inertia - Uterine inversion - Metrorrhagia in patient with uterine malformation	Microdebriefing during scenarios and post-simulation GAS debriefing.
6 Adult Emergency Clinical Teams 1 Emergency care physician, 2 Internists, 3 General practitioners, 12 Nurses, 4 Kinesiologists, 12 Paramedical technicians, 2 Support assistants.	6	- Ventricular fibrillation - Asystole - Pulseless electrical activity - Ventricular fibrillation	Microdebriefing during scenarios and post-simulation GAS debriefing.

In the case of clinical teams, simulations were developed in the framework of the Clinical Team Training Program of the Padre Hurtado Hospital, in training for the management of trauma in adolescents for Pediatric Emergency clinical teams, in training for the management of postpartum metrorrhagia for Obstetric Emergency clinical teams, and in the advanced cardiopulmonary resuscitation training program for adult Emergency teams during the second semester of 2021. Each training involved the outgoing clinical teams on duty, consisting of 6–8 members of the respective clinical units, each acting in his or her role. The teams tackled four simulation cases related to the thematic axis of their unit's program (Table 1). The scenarios were guided face-to-face by two instructors (a physician and a nurse or midwife), using a SimMom™ simulator with a vital signs monitor controlled with the LLEAP™ software or an ALS Laerdal™ simulator with a vital signs monitor controlled with SIMPAD™. The scenarios were implemented using the pause-and-repeat cycles recommended by deliberate rapid-cycle practice, with microdebriefing during the scenarios (20) and GAS debriefing following the scenarios. At the end of the session, the technicians and professionals who voluntarily agreed to participate in the study anonymously evaluated the teamwork of the last scenario, applying the TEAM scale supported by the SurveyMonkey™ platform. The reliability of the instrument was analyzed using Cronbach's alpha. We obtained approval from the Ethics Committee for Clinical Research of the Faculty of Medicine of the Universidad del Desarrollo (Act 2020-60).

## Results

In the process of cultural adaptation, we obtained a final instrument with 11 items on a 5-level Likert scale, grouped into three dimensions (leadership, teamwork, and task management), and a global appreciation scale from 1 to 10, as described in the original version (Supplementary Table 1). The scale was similar to that displayed on the original author's website, uploaded after our validation.

**Figure 1:**
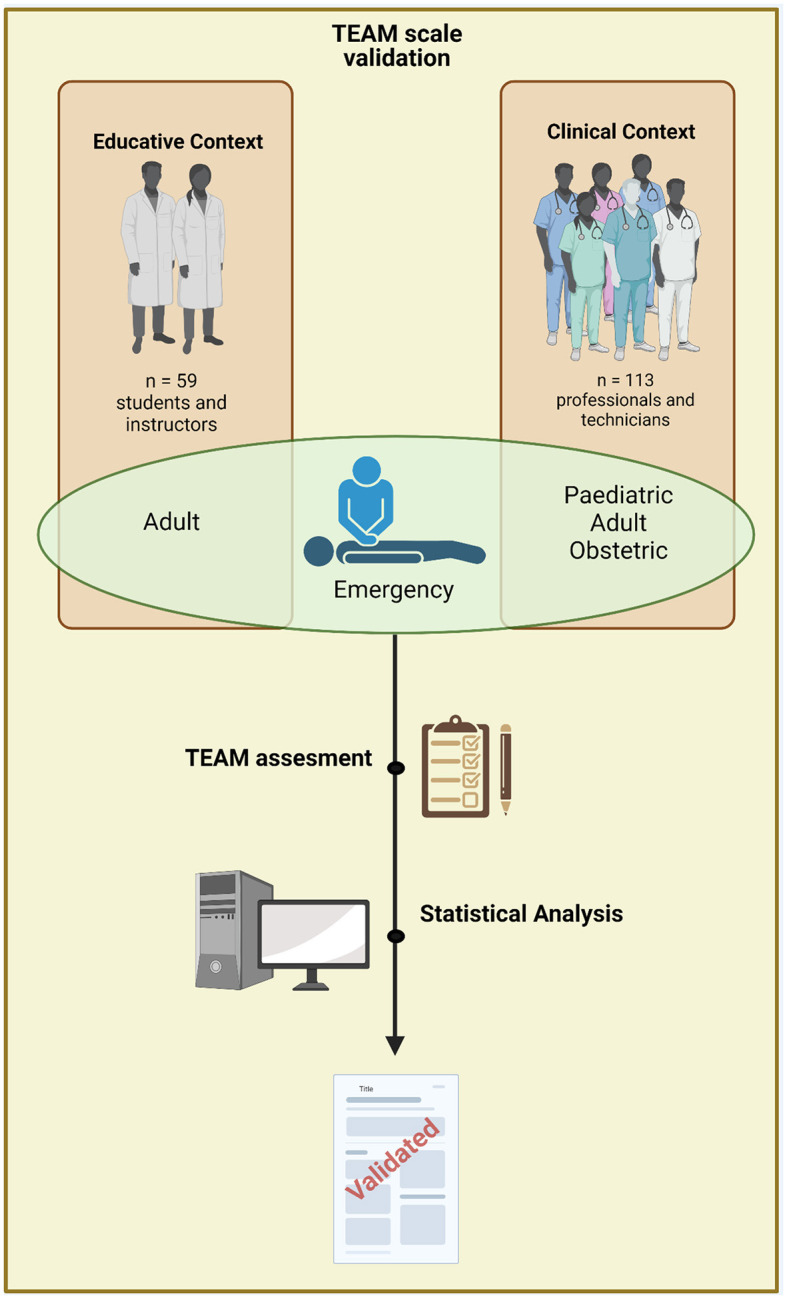
Flowchart of participants. Created with BioRender.com.

**Figure 2:**
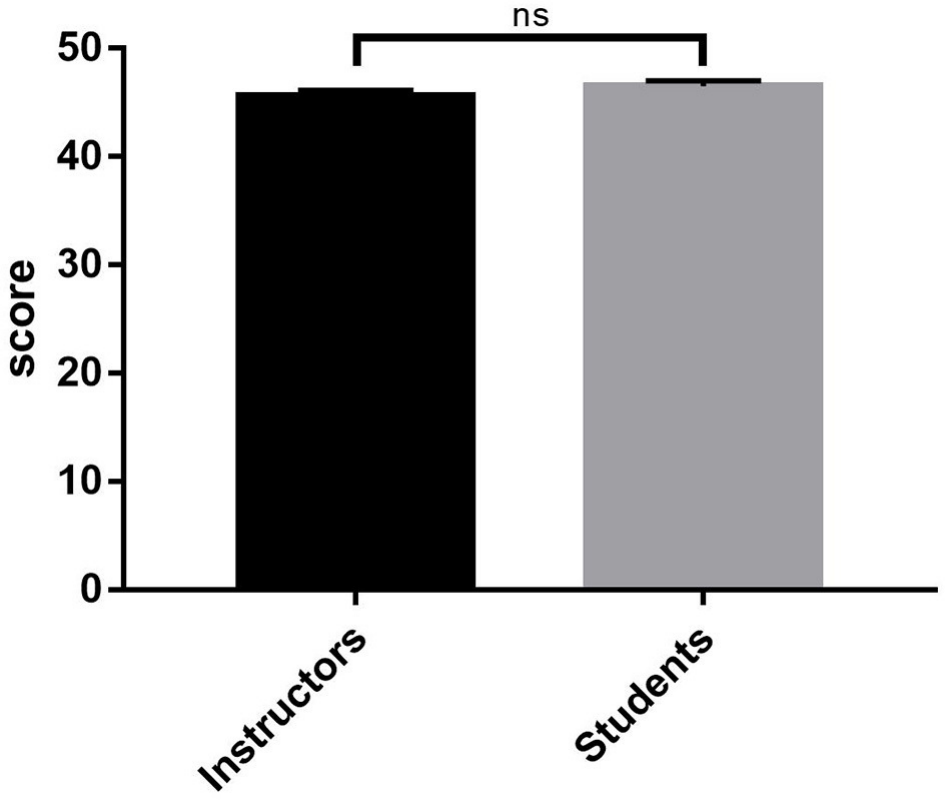
Overall score comparison in Spanish version of TEAM scale between instructors and students. ns, no significant.

For the validation in the context of training medical students, out of 82 students, 59 (72%) agreed to participate voluntarily in the instrument validation by answering the same (Figure 1). In all, 56% of the student volunteers were women. In evaluating the internal consistency of the instrument as a self-assessment tool for medicine students, Cronbach's alpha was 0.869. We analyze teamwork from the point of view of participating students (self-assessment) and instructors (professors), where the comparison of the overall results (Figure 2) did not show statistically significant differences (*p* = 0.119).

**Figure 3:**
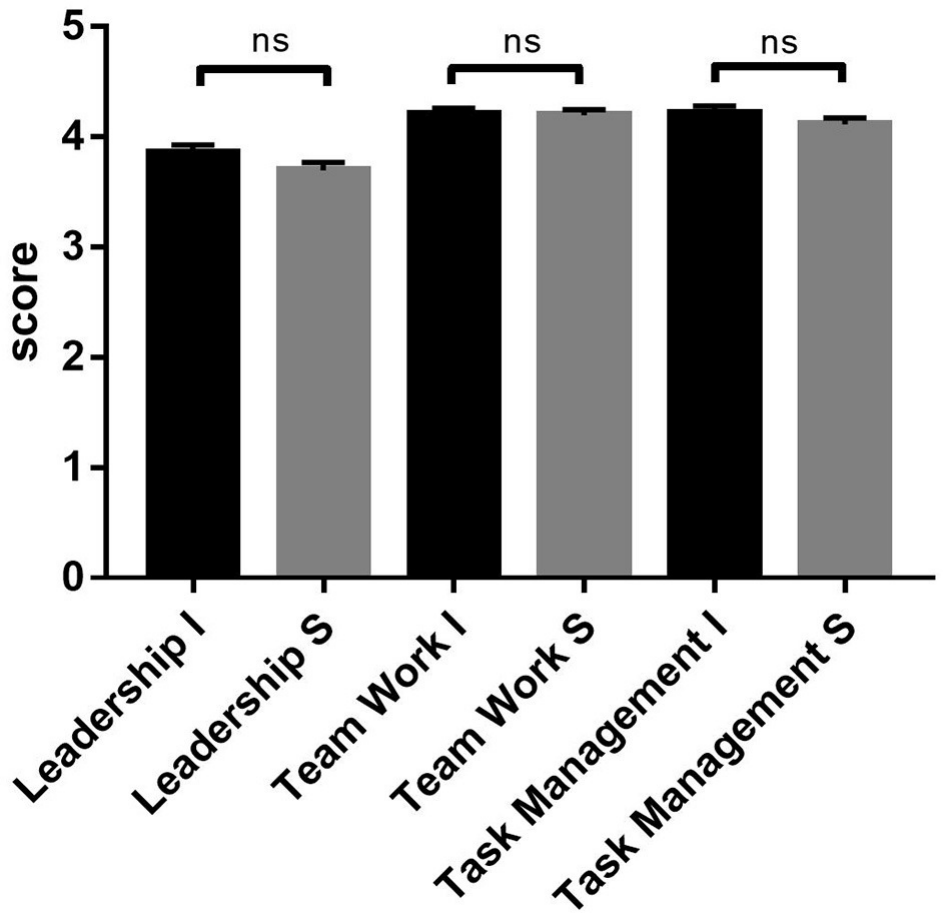
Subscale score observed by instructors (I) and students (S) in TEAM scale comparison. ns, no significant.

When evaluating the behavior of the items that make up the scale (Figure 3), in a disaggregated manner, both for the leadership (*p* = 0.193), teamwork (*p* = 0.646), and task management (*p* = 0.390) items, there were no statistically significant differences between instructors and students (significance *p* > 0.05).

Of the participating clinical teams belonging to Hospital Padre Hurtado, the Pediatric Emergency Department has 70 people, of whom 59 are women and 11 are men; the Obstetric Emergency Department has 83 people, of whom 73 are women and 10 are men; and the Adult Emergency Department has 123 members, including professionals and technicians, 79 women and 44 men.

In the team training simulations, 56 people from the Pediatric Emergency Department participated (80%), 40 from the Obstetric Emergency Department (48%), and 36 from the Adult Emergency Department (29%) (Figure 1, Table 1).

In the clinical team training analysis, the instrument's internal consistency yielded Cronbach's alpha of 0.755 for adult emergency, 0.797 for pediatric emergency, and 0.853 for obstetric emergency teams.

For the global analysis of the application of the instrument, considering all the responses of instructors (professors), students (self-assessment), and clinical teams, Cronbach's alpha value of 0.921 was reached.

## Discussion

The Spanish version of the TEAM scale achieves high levels of reliability, close to those found in its original version (5), where the analysis of 56 videos with the performance of emergency teams evaluated by an instructor reached Cronbach's alpha of 0.97. However, when the number of evaluating instructors was increased (to three), the internal consistency remained high, with an alpha of 0.895. Our study reproduces these values by obtaining alpha values of 0.869 for medical students and 0.853 for obstetric emergency clinical teams, with lower but equally good values of 0.797 for pediatric emergency teams and 0.755 for Chilean adult emergency teams.

McKay et al. (21), analyzed the consistency in evaluating emergency team performance by comparing the TEAM instrument with another scale for evaluating teams (OSCAR). In particular, the TEAM scale proved to be reliable for the serial evaluation of assessors and showed a reasonably high correlation with the OSCAR scale for assessing equipment (21). However, they did not report internal consistency data for the scale.

In our study, the results showed no significant differences between instructors' and students' ratings for the overall instrument and the evaluation of the three dimensions of the instrument. These findings are described for the first time in the literature and support the idea that both instructors and undergraduate medical students can apply the Spanish version of the TEAM scale. Given that the evaluation of teamwork is a necessity in undergraduate curricula internationally (1, 3) and in Chile (2), it is important to have a validated instrument to strengthen the teaching and learning processes of leadership and teamwork competencies. This instrument offers the opportunity to apply 360-degree evaluations within the undergraduate simulations, and the fact that they can be self-applied by the students can generate a catalytic effect of the evaluation (22), enhancing competency development.

In this study, the TEAM scale also showed high evidence of validity applied to undergraduate, pediatric, adult, and obstetric emergency contexts and scenarios involving trauma, cardiovascular and infectious emergencies, and one of the main obstetric emergencies, ratifying what has been previously described in the Anglo-Saxon literature (8, 11, 15). Using an objective and validated instrument to support the undergraduate training of future professionals who are more aware of the dimensions linked to teamwork competence may favor the possibility that new generations transfer this competence to their professional practice in any of these contexts, which is an additional strength and offers projections for the measurement of transfer to various clinical contexts.

This study was conducted exclusively in face-to-face simulations, so the scope of the use of this instrument in remote simulation contexts is an area that requires exploration in the Latin American context. The use of the TEAM instrument to evaluate the dynamics of remote and virtual simulation-trained teams has proven to be feasible and useful (23), including in the evaluation of video-trained rural emergency teams in Thailand and Myanmar (24). Exploring the use of the tool in this context may make sense in our region, where remote simulation can offer training opportunities to students, technicians, and professionals who need access to face-to-face simulation-based training at their study and work sites.

A limitation of this study is that the sample that participated in the validation of the instrument for adult emergency represented 30% of the unit's workers. Adding new evidence in this context with a larger population may be necessary.

Evaluating the performance of undergraduate students, emergency technicians, and professionals with an instrument that has evidence of validity at a global level also offers an important global education tool for Chilean students, technicians, and professionals, which is highly relevant in a global healthcare context.

## Data availability statement

The raw data supporting the conclusions of this article will be made available by the authors, without undue reservation.

## Ethics statement

The studies involving human participants were reviewed and approved by Comité de Ética de la Investigación–Facultad de Medicina Universidad del Desarrollo. The patients/participants provided their written informed consent to participate in this study.

## Author contributions

LP: Data curation, Formal analysis, Writing—original draft, Writing—review and editing. SA-R: Conceptualization, Funding acquisition, Methodology, Validation, Writing—original draft, Writing—review and editing. SF-R: Investigation, Writing—review and editing. MA-T: Investigation, Writing—review and editing.
