# Supplementary material for: Application of the Team Emergency Assessment Measure Scale in undergraduate medical students and interprofessional clinical teams: validity evidence of a Spanish version applied in Chile
Source: Front Med (Lausanne). 2023 Sep 13;10:1256982. doi: 10.3389/fmed.2023.1256982 (PMC10525305; doi:10.3389/fmed.2023.1256982)
Supplement: Supplementary file 1 [file Table_1.docx]

**Supplementary Table 1: Spanish version of Team Emergency Measurement Scale (in spanish)**

| Instrucciones - Escala de Trabajo en Equipo en Urgencia (Team Emergency Assessment Measure Scale, Cooper 2010)  Esta escala fue diseñada para evaluar el desempeño de equipos de urgencia (equipos de reanimación y de trauma). La pauta fue diseñada para ser rellenada por clínicos expertos para permitir una calificación precisa del rendimiento y una retroalimentación sobre el liderazgo, el trabajo en equipo, el conocimiento de la situación y la gestión de las tareas por expertos en la materia. Se incluyen indicaciones de calificación cuando procede. | | | | |
| --- | --- | --- | --- | --- |
| 0 = Nunca/ casi nunca | 1 = Rara vez | 2 = Más o menos a menudo | 3 = A menudo | 4 = Siempre/casi siempre |

| **Liderazgo (se supone que el líder está designado, ha surgido o es el más veterano - si no surge ningún líder asigne un 0 a las preguntas 1 y 2)** | **0** | **1** | **2** | **3** | **4** |
| --- | --- | --- | --- | --- | --- |
| 1.- El líder del equipo hizo saber al equipo lo que se esperaba de ellos, mediante la conducción y entrega de instrucciones claras |  |  |  |  |  |
| 2.- El líder del equipo mantuvo una perspectiva global  (Indicación de calificación: ¿controla los procedimientos clínicos y el entorno? ¿se mantiene sin intervenir, según el caso? ¿delega adecuadamente las tareas?) |  |  |  |  |  |
| **Trabajo en equipo: Las calificaciones deben incluir al equipo en su conjunto, es decir, al líder y al equipo como colectivo (en mayor o menor medida)** | **0** | **1** | **2** | **3** | **4** |
| 3.- El equipo se comunicó eficazmente  (Indicación de calificación: ¿formas de comunicación verbal, no verbal y escrita?) |  |  |  |  |  |
| 4.- El equipo trabajó conjuntamente para completar las tareas en el momento oportuno |  |  |  |  |  |
| 5.- El equipo actuó con compostura y control  (Indicación de calificación: ¿emociones relevantes? ¿cuestiones de gestión de conflictos? |  |  |  |  |  |
| 6.- La moral del equipo fue positiva  (Indicación de calificación: ¿apoyo adecuado, confianza, espíritu, optimismo, determinación? |  |  |  |  |  |
| 7.- El equipo se adaptó a las situaciones cambiantes  (Indicación de calificación: ¿adaptación dentro de las funciones de su profesión?. Cambios de situación ¿deterioro del paciente? ¿cambios dentro del equipo? |  |  |  |  |  |
| 8.- El equipo supervisó y reevaluó la situación |  |  |  |  |  |
| 9.- El equipo se anticipó a las posibles acciones  (Indicación de calificación: ¿preparación del desfibrilador, de los fármacos, del equipo de la vía aérea? |  |  |  |  |  |
| **Manejo de la tarea** | **0** | **1** | **2** | **3** | **4** |
| 10.- El equipo priorizó las tareas |  |  |  |  |  |
| 11.- El equipo siguió las normas/directrices aprobadas  (Indicación de calificación: algunas desviaciones pueden ser apropiadas) |  |  |  |  |  |
| Apreciación global | 1-2 | 3 - 4 | 5 - 6 | 7 - 8 | 9 - 10 |
| 12.- En una escala de 1 a 10 indique su apreciación global del equipo |  |  |  |  |  |
